# Genotypic distribution and molecular spectrum of rare and novel thalassemia variants in Ganzhou, southern China

**DOI:** 10.3389/fmolb.2026.1833133

**Published:** 2026-09-03

**Authors:** Jungao Huang, Xiaoyan Huang, Xinxing Xie, Xiaoqin Xin

**Affiliations:** 1 Ganzhou Maternal and Child Health Hospital, Ganzhou, Jiangxi, China; 2 BGI-Wuhan, Shenzhen, China; 3 Department of Clinical Laboratory, Ganzhou People’s Hospital, Ganzhou, Jiangxi, China

**Keywords:** general population, next-generation sequencing (NGS), novel, rare, thalassemia

## Abstract

**Background:**

Thalassemia is the most common single-gene inherited blood disorder worldwide, being relatively rare in northern China but more prevalent in southern China. To facilitate effective prevention and control of thalassemia, we analyze the genotype and frequency distribution of rare thalassemia variants in the general population of the Ganzhou area, offering valuable insights for genetic counseling and prenatal diagnosis.

**Methods:**

Between January 2022 and January 2025, a cohort of 84,067 individuals was screened in Ganzhou. Following the exclusion criteria, 1,081 participants with rare thalassemia variants were included in the final analysis. Genotypes associated with thalassemia were determined using next-generation sequencing (NGS). Variant classification adhered to ACMG/AMP guidelines.

**Results:**

Among the participants with rare thalassemia variants, 495 individuals presented with rare α-thalassemia variants and 560 with rare β-thalassemia variants, including 26 newly identified variants. Rare thalassemia variants accounted for 1.29% of the total screened population. The distribution of rare thalassemia variants exhibited regional variation across Ganzhou, with the highest rates in Nan Kang (12.69%), followed by Xingguo (10.49%) and Zhanggong (8.80%). Chongyi had the lowest rate at 1.78%, with all these locations located to the northwest of Ganzhou. Additionally, 26 individuals carried novel thalassemia variants. Six of these variants were classified as “Likely Pathogenic,” and the HBB:c.50G>T (Gly > Val) genotype was associated with hypochromic anemia.

**Conclusion:**

This study offers a comprehensive analysis of rare and newly identified thalassemia variants in the Ganzhou area, highlighting the complexity and heterogeneity of the condition. These findings underscore the necessity for effective screening methods in regions with a high incidence of thalassemia and provide essential insights for the targeted prevention and management of this disorder in the future.

## Introduction

Thalassemia is a genetic hematological disorder characterized by insufficient hemoglobin production, affecting millions of individuals worldwide. Approximately 5.2% of the global population is afflicted, making it one of the most prevalent single-gene diseases (Tarim and Oz, 2022). Each year, between 300,000 and 400,000 infants are born with various forms of thalassemia, underscoring the significant global health burden associated with this condition (Vachhani et al., 2022). In China, the prevalence of thalassemia is particularly high in the southern regions, especially south of the Yangtze River, where carrier rates rank among the highest in the country (Wang WD. et al., 2023). The Ganzhou area, known for its elevated incidence of thalassemia (Zhao et al., 2018), is home to a significant population of Hakka people, exhibiting an alarming carrier rate of 14.54% (Xie et al., 2023).

Clinically, thalassemia primarily manifests as hypochromic microcytic hemolytic anemia and is often accompanied by symptoms such as splenomegaly, jaundice, and characteristic skeletal deformities (Ansharullah et al., 2025). Thalassemia can be categorized into several types, including α-, β-, δ-, and γ-thalassemia, with α- and β-thalassemia being the two predominant forms encountered in clinical practice. These conditions arise from the loss or mutation of the β-globin gene (HBB) or the α-globin genes (HBA1 and HBA2), respectively (Zhang H. et al., 2025). The severity of clinical manifestations and anemia varies based on the specific genetic variant. While thalassemia minor may present as mild anemia or remain asymptomatic, thalassemia major typically results in severe anemia (Zakaria et al., 2021).

Despite advancements in therapies such as blood transfusions, bone marrow transplants, and gene therapy, prevention remains the most effective and straightforward strategy (Hu et al., 2024; Laurent et al., 2024; Philippidis, 2024). Current preventive measures primarily utilize a three-tier screening approach, encompassing routine blood tests, hemoglobin electrophoresis, and genetic confirmation (Fakher et al., 2007; Li et al., 2023). However, conventional testing mainly targets 23 common variants—three mutation types and three deletions for α-thalassemia and 17 mutations for β-thalassemia—thus leaving many rare variants undetected and complicating prevention efforts. To date, over 1,000 thalassemia variants have been documented in the specialized globin gene server database, HbVar (https://globin.bx.psu). Notably, these conventional targeted approaches continue to be widely used in routine screening and diagnostic settings, owing to their established reliability, low bioinformatics demands, and straightforward scalability for population-level screening.

Recent advancements in genomic technologies, particularly next-generation sequencing (NGS), have improved the detection of rare thalassemia variants. Previous studies indicate that direct NGS could reduce misdiagnosis rates by 30.5%–74.8% compared to traditional genetic screening methods (He et al., 2017; Yang et al., 2023; Cao et al., 2026). However, many earlier studies did not compile a comprehensive catalog of rare thalassemia cases, and it remains uncertain whether the prevalence of these rare variants varies across different regions.

To enhance thalassemia prevention efforts, we employed NGS techniques in the Ganzhou area to identify rare and newly emerging thalassemia variants. This initiative aims to inform and improve future governmental health policies. Our study involves a substantial cohort of 84,067 participants, spanning from January 2022 to January 2025, laying a robust theoretical foundation for thalassemia screening, targeted prevention strategies, and effective interventions.

## Participants and methods

**FIGURE 1:**
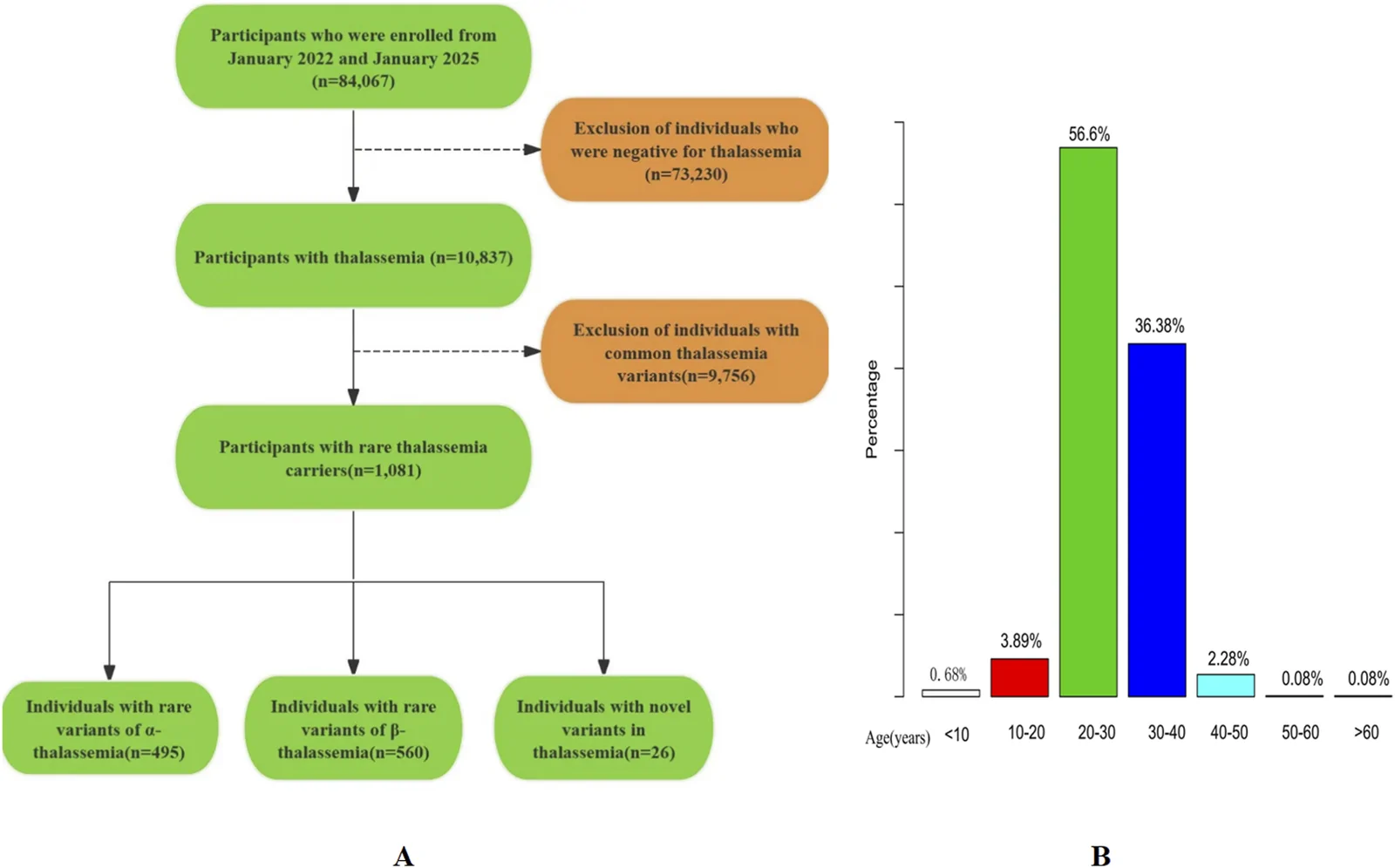
The population in the study. **(A)** Flow chart of the enrollment of participants. **(B)** Constituent ratios of different age groups of 1081 participants carried rare/novel thalassemia variants.

This study involved a cohort of 84,067 individuals screened in Ganzhou, Southern China, from January 2022 to January 2025. After excluding non-thalassemia carriers (n = 73,230), 10,837 participants with thalassemia variants remained. Further exclusion of those with variants detectable by traditional methods (n = 9,756) narrowed the final analysis to 1,081 participants with rare variants (Figure 1A). The majority of participants were local residents from various districts of Ganzhou, predominantly Han (99.1%), with a minority representing Hui and She ethnic groups (0.9%). Age distribution ranged from 1 to 87 years, with a mean age of 27 years. All individuals provided written informed consent prior to participation. Any variant identified by our NGS assay that fell outside the routine 23-variant panel was classified as “rare”. Variants that have not been previously described were classified as “novel”.

Blood samples were collected from participants and analyzed using an automated blood analyzer (UniCel DxH 800; Beckman, United States) to assess key hematological parameters, including hemoglobin concentration, mean corpuscular volume (MCV), and mean corpuscular hemoglobin (MCH). Participants were categorized as positive if MCH was <27 pg and/or MCV was <82 fL. Genomic DNA was extracted from peripheral blood samples using the QIAamp Blood Mini Kit (Qiagen; Hilden, Germany), following the manufacturer’s protocol. The purity and concentration of the extracted DNA were measured using a spectrophotometer (NanoDrop 2000; Thermo Scientific, United States), with only samples having concentrations >20 ng/mL and an A260/A280 ratio between 1.8 and 2.0 accepted for analysis.

The sequencing method used was consistent with our previous reports (Xie et al., 2023). Briefly, full-length amplification of the HBA1, HBA2, and HBB genes was performed via polymerase chain reaction (PCR), ensuring coverage of all relevant exons and introns identified in the HbVar database (https://globin.bx.psu). The primer panel comprised six pairs of oligonucleotides, targeting four genes (HBA1, HBA2, HBB-1, and HBB-2) and two common deletion breakpoints (HBA-Q and HBB-Q). The PCR amplification was carried out in 96-well plate format, with each well corresponding to an individual sample library. A set of 96 unique index sequences was designed and assigned to each well, and the six target-specific primers labeled with these index sequences were designated as index primers, enabling sample barcoding and multiplex sequencing in a single run. The primers are associated with patents WO/2014/023076, WO/2014/023167, and CN102952877. NGS libraries were prepared according to the Illumina HiSeq Sequencing Library Preparation Protocol, and sequencing was conducted on the Illumina HiSeq2000 platform using paired-end sequencing for 100 base pairs. Subsequent bioinformatics analyses included variant calling and annotation, employing established methodologies to ensure accurate identification of genetic variants. Variants identified by NGS were validated by Sanger sequencing, which ensured the accuracy and reliability of all reported rare and novel variants.

Common deletions associated with α-thalassemia or β-thalassemia were further validated using Gap-PCR (BGI, Shenzhen, China) and 4% agarose gel electrophoresis. Variant interpretation and classification followed the gene-specific ACMG/AMP guidelines established by the ClinGen Hemoglobinopathy Variant Curation Expert Panel (Kountouris et al., 2022).

### Statistical analysis

All statistical analyses were performed using SPSS version 27.0 software (SPSS Inc., Chicago, IL, United States). Categorical variables are reported as counts (n) and percentages (%). Chi-square tests were employed to compare the detection rates of α-thalassemia and β-thalassemia. A two-sided P-value of <0.05 was considered statistically significant.

## Results

A total of 1,081 participants with rare thalassemia variants were included in the final analysis. Among these, 495 individuals exhibited rare α-thalassemia variants, while 560 carried rare β-thalassemia variants, including 26 individuals with newly identified variants. Overall, rare thalassemia variants accounted for 1.29% of the total screened population (1,081 out of 84,067) (Figure 1A).

**FIGURE 2:**
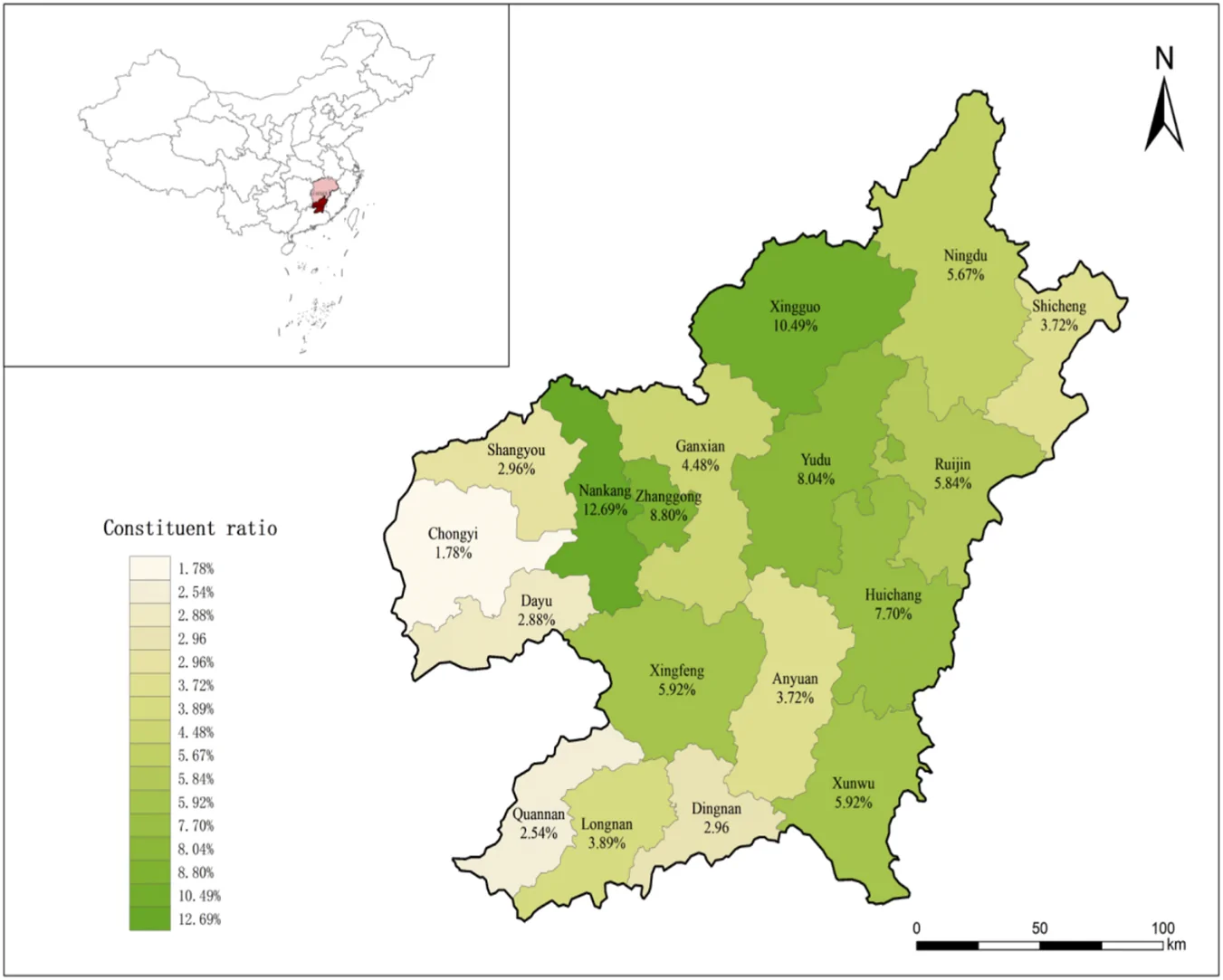
Geographic distribution of rare thalassemia in Southern Jiangxi, China.

In terms of age distribution, the 20–30 age group comprised 56.6% of participants, followed by 36.38% in the 30–40 age bracket, indicating a predominance of individuals of reproductive age (Figure 1B). The distribution of rare thalassemia variants displayed regional variation across Ganzhou, with the highest rates observed in Nan Kang (12.69%), followed by Xingguo (10.49%) and Zhanggong (8.80%). Chongyi recorded the lowest rate at 1.78%. Notably, all these locations are situated to the northwest of Ganzhou (Figure 2).

Clinically, α/β thalassemia variants are categorized as severe, and mild (α0, and α+; β0, and β+). Among the 495 individuals with rare α-thalassemia variants, we identified 41 distinct types, with Hb Hekinan II (HBA1:c.84G>T) (α+) being the most prevalent at 28.283%, corresponding to a population frequency of 0.167% (140 out of 84,067). The αα/--THAI (α0) variant constituted 3.030%. The top nine genotypes of rare α-thalassemia accounted for the majority, representing 86.06% of the cases (Table 1). Among the 560 individuals with rare β-thalassemia, we identified 44 types, with the 32019UTR +129 (T>A) (β+) variant being the most common at 27.679%, translating to a frequency of 0.184%. The β0 variant with the highest occurrence was (SEA)-HPFH, observed at a rate of 2.857%. Similarly, the top nine genotypes of rare β-thalassemia represented the majority, comprising 83.93% of the total (Table 2).

**TABLE 1:** 41 rare variants of α-thalassemia, and their frequency in the population.

Category	Rare variants	HGVS (Nucleotide)	HGVS (Protein)	Class	Number(n)	Ratio (%)	Frequency (%)
1	Hb Hekinan II	NM_000558.5:c.84G>T	p.Trp28Cys	α+	140	28.283%	0.167%
2	Hb Q-Thailand	NM_000558.5:c.223G>C	p.Asp75His	Hb variant	73	14.747%	0.087%
3	Hb Owari	NM_000558.5:c.364G>A	p.Asp122Asn	Hb variant	45	9.091%	0.054%
4	Hb G-Honolulu	NM_000517.6:c.91G>C	p.Asp31His	Hb variant	43	8.687%	0.051%
5	Hb Nanchang	NM_000517.6:c.46G>A	p.Gly16Ser	Hb variant	32	6.465%	0.038%
6	Hb Binyang	NM_000517.6:c.40G>T	p.Ala14Ser	Hb variant	28	5.657%	0.033%
7	HKαα	NG_000006.1:g.?_?	—	α+	26	5.253%	0.031%
8	Hb Phnom Penh	NG_000006.1:g.?_?	—	α+	24	4.848%	0.029%
9	αα/--THAI	NG_000006.1:g.10664_44164del33501	—	α0	15	3.030%	0.018%
10	Initiation codon (ATG>A-G)	NM_000558.5:c.2T>G	p.Met1Arg	α+	13	2.626%	0.015%
11	Hb Hengqin I	NM_000558.5:c.16G>A	p.Gly6Ser	Hb variant	6	1.212%	0.007%
12	Hb Liaobu	NM_000517.6:c.322G>C	p.Asp108His	Hb variant	4	0.808%	0.005%
13	Hb Hollythorpe	NM_000517.6:c.96-12T>A	Non-coding (intronic)	Hb variant	4	0.808%	0.005%
14	Hb Venetia	NM_000517.6:c.65C>T	p.Pro22Leu	Hb variant	3	0.606%	0.004%
15	Hb Ube-2	NM_000558.5:c.205A>G	p.Asn69Asp	Hb variant	3	0.606%	0.004%
16	Hb Ottawa	NM_000517.6:c.46G>C	p.Gly16Arg	Hb variant	3	0.606%	0.004%
17	Alpha2 Codon 30 del GAG	NM_000517.6:c.91_93delGAG	p.Glu31del	α+	3	0.606%	0.004%
18	α2 Codon 31 (AGG>AAG)	NM_000517.6:c.94G>A	p.Glu32Lys	α+	2	0.404%	0.002%
19	Codon 61 (AAG>TAG) [Lys > STOP]	NM_000558.5:c.184A>T	p.Lys62Ter	α+	2	0.404%	0.002%
20	Codon 22 (GGC>GGT)	NM_000517.6:c.67G>T	p.Gly23Cys	α+	2	0.404%	0.002%
21	Hb Zurich-Albisrieden	NM_000558.5:c.178G>C	p.Ala60Pro	α+	2	0.404%	0.002%
22	Hb Port Phillip	NM_000517.6:c.275T>C	p.Leu92Pro	Hb variant	2	0.404%	0.002%
23	Hb Groene Hart	NM_000558.5:c.358C>T	p.Pro120Ser	α+	2	0.404%	0.002%
24	IVS-I-38 (C>T)	NM_000517.6:c.95-38C>T	Non-coding (intronic)	α+	1	0.202%	0.001%
25	IVS-I-116 (A>G)	NM_000517.6:c.95-116A>G	Non-coding (intronic)	α+	1	0.202%	0.001%
26	IVS-I-1 (AGGT>AGAT) donor	NM_000517.6:c.95+1G>A	Non-coding (intronic)	α+	1	0.202%	0.001%
27	Hb Xuchang	NM_000517.6:c.133C>T	p.Pro45Ser	Hb variant	1	0.202%	0.001%
28	Hb St. Luke’s	NM_000558.5:c.287C>G	p.Pro96Arg	Hb variant	1	0.202%	0.001%
29	Hb Northwood	NM_000558.5:c.293A>G	p.Asn98Ser	Hb variant	1	0.202%	0.001%
30	Hb Macarena	NM_000517.6:c.358C>T	p.Pro120Ser	Hb variant	1	0.202%	0.001%
31	Hb Luxembourg	NM_000558.5:c.73T>C	p.Trp25Arg	Hb variant	1	0.202%	0.001%
32	Hb Liuzhou-Yufeng	NM_000558.5:c.334G>T	p.Asp112Tyr	Hb variant	1	0.202%	0.001%
33	Hb J-Toronto	NM_000558.5:c.17C>A	p.Ala6Asp	Hb variant	1	0.202%	0.001%
34	Hb J-Tashikuergan	NM_000558.5:c.59C>A	p.Ala20Asp	Hb variant	1	0.202%	0.001%
35	Hb Garden state	NM_000558.5:c.248C>A	p.Pro83GLn	Hb variant	1	0.202%	0.001%
36	Hb Dagestan	NM_000558.5:c.181A>G	p.Lys61GLu	Hb variant	1	0.202%	0.001%
37	Hb Broomhill	NM_000558.5:c.343C>G	p.Pro115Ala	Hb variant	1	0.202%	0.001%
38	Fusion gene	—	—	α+	1	0.202%	0.001%
39	Codons 90–92 (-8bp): (-AGCTTCGG)	NM_000558.5:c.270_277delAGCTTCGG	p.?	α+	1	0.202%	0.001%
40	Hb Amsterdam-A1	NM_000558.5:c.99G>A	p.Trp33Ter	α+	1	0.202%	0.001%
41	Hb Dapu	NM_000517.6:c.52G>T	p.Ala18Ser	Hb variant	1	0.202%	0.001%
Total					495	100.000%	0.589%

**TABLE 2:** 44 rare variants of β-thalassemia, and their frequency in the population.

Category	Rare variants	HGVS (Nucleotide)	HGVS (Protein)	Class	Number(n)	Ratio (%**)**	Frequency (%**)**
1	3′UTR +129 (T>A)	NM_000518.5:c.*129T>A	Non-coding (3′UTR)	β+	155	27.679%	0.184%
2	−50 (G>A)	NM_000518.5:c.-50G>A	Non-coding (5′UTR)	β+	71	12.679%	0.084%
3	Hb New York	NM_000518.5:c.341T>A	p.Val114GLu	Hb variant	57	10.179%	0.068%
4	Chinese Gγ+(Aγδβ)0	NG_000007.3:g.48795_127698del78904	—	β0	47	8.393%	0.056%
5	Hb Zengcheng	NM_000518.5:c.343C>A	p.Pro115Thr	Hb variant	46	8.214%	0.055%
6	Hb J-Bangkok	NM_000518.5:c.170G>A	p.Gly57Asp	Hb variant	39	6.964%	0.046%
7	−63 (A>G)	NM_000518.5:c.-63A>G	Non-coding (5′UTR)	β+	30	5.357%	0.036%
8	(SEA)-HPFH	NG_000007.3:g.48795_127698del78904	—	β0	16	2.857%	0.019%
9	−90 (C>T)	NM_000518.5:c.-90C>T	Non-coding (5′UTR)	β+	9	1.607%	0.011%
10	Hb Zurich-Langstrasse	NM_000518.5:c.151A>T	p.Met51Leu	Hb variant	8	1.429%	0.010%
11	Codons 54–58 (-TATGGGCAACCCT)	NM_000518.5:c.165_177delTATGGGCAACCCT	p.Leu19_Leu20del?	β0	7	1.250%	0.008%
12	Taiwanese deletion	NG_000007.3:g.48795_127698del78904	—	β0	6	1.071%	0.007%
13	CAP +8 (C>T)	NM_000518.5:c.-8C>T	Non-coding (5′UTR)	β+	6	1.071%	0.007%
14	−72 (T>A)	NM_000518.5:c.-72T>A	Non-coding (5′UTR)	β+	6	1.071%	0.007%
15	−42 (-C)	NM_000518.5:c.-42delC	Non-coding (5′UTR)	β+	6	1.071%	0.007%
16	5′UTR +22 (G>A)	NM_000518.5:c.-15G>A	Non-coding (5′UTR)	β+	5	0.893%	0.006%
17	Hb Molfetta	NM_000518.5:c.379G>C	p.Asp127His	Hb variant	5	0.893%	0.006%
18	Hb J-Taichung	NM_000518.5:c.389C>A	p.Ala130Asp	Hb variant	5	0.893%	0.006%
19	Hb G-Siriraj	NM_000518.5:c.22G>A	p.Gly8Ser	Hb variant	4	0.714%	0.005%
20	Hb Hamilton	NM_000518.5:c.34G>A	p.Gly12Arg	Hb variant	3	0.536%	0.004%
21	Hb G-Taipei	NM_000518.5:c.68A>G	p.Asp23GLy	Hb variant	3	0.536%	0.004%
22	Hb Taipei-Tien	NM_000518.5:c.122G>T	p.Gly41Val	Hb variant	3	0.536%	0.004%
23	Hb Renert	NM_000518.5:c.401T>C	p.Val134Ala	Hb variant	2	0.357%	0.002%
24	Cap +1570 (T>C)	NM_000518.5:c.*1570T>C	Non-coding	β+	1	0.179%	0.001%
25	5′UTR +28 (A>G)	NM_000518.5:c.-9A>G	Non-coding (5′UTR)	β+	1	0.179%	0.001%
26	3′UTR +132 (C>T)	NM_000518.5:c.*132C>T	Non-coding (3′UTR)	β+	1	0.179%	0.001%
27	3′UTR +118 (A>G)	NM_000518.5:c.*118A>G	Non-coding (3′UTR)	β+	1	0.179%	0.001%
28	−32 (C>T)	NM_000518.5:c.-32C>T	Non-coding (5′UTR)	β+	1	0.179%	0.001%
29	Hb Dhonburi	NM_000518.5:c.380T>G	p.Leu127Arg	β+	1	0.179%	0.001%
30	Hb Cochin-Port Royal	NM_000518.5:c.440A>G	p.Asp147GLy	Hb variant	1	0.179%	0.001%
31	IVS-I (−2) or codon 30 (A>G)	NM_000518.5:c.30A>G	Non-coding (intronic)	β0	1	0.179%	0.001%
32	Poly A (AATAAA>AATGAA)	NM_000518.5:c.*110T>C	Non-coding (3′UTR)	β+	1	0.179%	0.001%
33	IVS-II-63 (T>C)	NM_000518.5:c.316-63T>C	Non-coding (intronic)	β+	1	0.179%	0.001%
34	IVS-II-613 (C>T)	NM_000518.5:c.316-613C>T	Non-coding (intronic)	β+	1	0.179%	0.001%
35	IVS-I-129 (A>G)	NM_000518.5:c.129A>G	Non-coding (intronic)	β0	1	0.179%	0.001%
36	Hb Bologna-St.Orsola	NM_000518.5:c.439C>T	p.Pro147Ser	Hb variant	1	0.179%	0.001%
37	Hb Alperton	NM_000518.5:c.407C>T	p.Pro136Leu	Hb variant	1	0.179%	0.001%
38	Hb City of Hope	NM_000518.5:c.208G>A	p.Gly70Ser	Hb variant	1	0.179%	0.001%
39	Hb Kofu	NM_000518.5:c.254C>T	p.Pro85Leu	Hb variant	1	0.179%	0.001%
40	Hb Sitia	NM_000518.5:c.386C>T	p.Pro129Leu	Hb variant	1	0.179%	0.001%
41	Hb Rambam	NM_000518.5:c.209G>A	p.Gly70Asp	Hb variant	1	0.179%	0.001%
42	Hb Midnapore	NM_000518.5:c.161C>T	p.Pro54Leu	Hb variant	1	0.179%	0.001%
43	Hb Pierre-Bénite	NM_000518.5:c.273G>C	p.Lys91Asn	Hb variant	1	0.179%	0.001%
44	Hb Olympia	NM_000518.5:c.61G>A	p.Gly21Ser	Hb variant	1	0.179%	0.001%
Total					560	100.000%	0.666%

Additionally, 26 individuals exhibited novel thalassemia variants, totaling 23 distinct types, all in a heterozygous state. Six of these variants were classified as “Likely Pathogenic”: HBA1:c.223G>T (p.Asp75Tyr), HBA1:c.268C>G (p.His90Asp), HBA2:c.63C>G (p.His21GLn), HBB:c.-159delA (non-coding, 5‘UTR), HBB:c.316-3C>T (non-coding, intronic), and HBB:c.50G>T (p.Gly17Val). The remaining variants were categorized as “Variants of Uncertain Significance” (VUS) (Table 3). Hematological evaluations revealed that individuals with the HBB:c.50G>T (p.Gly17Val) genotype presented with hypochromic anemia, whereas the clinical phenotypes of the other genotypes did not exhibit significant alterations (Table 4).

**TABLE 3:** The distrubition of 23 novel variants in thalassemia.

Category	Novel variants	Amino acid change (HGVS)	Status	Number of cases	Male	Female	Pathogenity
1	HBA1:c.*13_14insT	Non-coding (3‘ UTR)	Heterozygous	1	1	-	VUS
2	HBA1:c.*35delG	Non-coding (3‘ UTR)	Heterozygous	1	-	1	VUS
3	HBA1:c.*57delC	Non-coding (3‘ UTR)	Heterozygous	1	-	1	VUS
4	HBA1:c.*74_75insC	Non-coding (3‘ UTR)	Heterozygous	2	-	2	VUS
5	HBA1:c.*94A>C	Non-coding (3‘ UTR)	Heterozygous	1	-	1	VUS
6	HBA1:c.*95A>T	Non-coding (3‘ UTR)	Heterozygous	1	1	-	VUS
7	HBA1:c.202A>G	p.Thr68Ala	Heterozygous	1	-	1	VUS
8	HBA1:c.223G>T	p.Asp75Tyr	Heterozygous	1	-	1	Likely pathogenic
9	HBA1:c.268C>G	p.His90Asp	Heterozygous	1	-	1	Likely pathogenic
10	HBA2:c.*91_92insA	Non-coding (3’ UTR)	Heterozygous	1	-	1	VUS
11	HBA2:c.118A>G	p.Thr40Ala	Heterozygous	1	-	1	VUS
12	HBA2:c.300 + 43delG	Non-coding (intronic)	Heterozygous	1	1	-	VUS
13	HBA2:c.301-35delG	Non-coding (intronic)	Heterozygous	2	1	1	VUS
14	HBA2:c.63C>G	p.His21GLn	Heterozygous	1	1	-	Likely pathogenic
15	HBA2:c.95 + 11delC	Non-coding (intronic)	Heterozygous	1	-	1	VUS
16	HBA2:c.95+9C>G	Non-coding (intronic)	Heterozygous	1	-	1	VUS
17	HBB:c.*145delA	Non-coding (3‘ UTR)	Heterozygous	1	-	1	VUS
18	HBB:c.-159delA	Non-coding (5’ UTR)	Heterozygous	1	-	1	Likely pathogenic
19	HBB:c.-28T>C	Non-coding (5′ UTR)	Heterozygous	1	-	1	VUS
20	HBB:c.316-3C>T	Non-coding (intronic)	Heterozygous	2	-	2	Likely pathogenic
21	HBB:c.-49C>T	Non-coding (5′ UTR)	Heterozygous	1	-	1	VUS
22	HBB:c.50G>T	p.Gly17Val	Heterozygous	1	-	1	Likely pathogenic
23	HBB:c.93-53_93-52insTTC	Non-coding (intronic)	Heterozygous	1	-	1	VUS
Total				26	5	21	

VUS: variant of uncertain signifcance; -: NA, Variant annotations were performed using the following reference transcript sequences: HBA1: NM_000558.5, HBA2: NM_000517.6, and HBB: NM_000518.5.

**TABLE 4:** The phenotypes of 6 novel variants which predicted to be pathogenic.

Novel variants	Amino acid change (HGVS)	Gender	Age (years)	RBC (1012**/L)**	MCV(fl)	MCH(pg)	Hb(g/dL)
HBA1:c.223G>T	p.Asp75Tyr	Female	42	4.49	85.5	29.8	134
HBA1:c.268C>G	p.His90Asp	Female	30	5.04	87.9	28.3	143
HBA2:c.63C>G	p.His21GLn	Male	26	5.18	82.3	29.8	155
HBB:c.-159delA	Non-coding (5′ UTR)	Female	30	4.71	85.8	29.4	138
HBB:c.316-3C>T	Non-coding (intronic)	Female	31	3.74	88.4	29.4	110
HBB:c.316-3C>T	Non-coding (intronic)	Female	24	4.17	94.1	28.9	121
HBB:c.50G>T	p.Gly17Val	Female	24	5.31	66.6	19.2	102

MCV: mean corpuscular volume; MCH: mean corpuscular hemoglobin; Hb: Hemoglobin. Variant annotations were performed using the following reference transcript sequences: HBA1: NM_000558.5, HBA2: NM_000517.6, and HBB: NM_000518.5.

## Discussion

The global public health burden of thalassemia, a group of inherited blood disorders, is significant due to its high prevalence and diverse clinical manifestations (Tuo et al., 2024). Most thalassemia carriers can typically be identified through standard hematological analyses of mean corpuscular volume (MCV) and mean corpuscular hemoglobin (MCH), supplemented by traditional genetic testing. Methods for detecting thalassemia include restriction fragment length polymorphism (RFLP), polymerase chain reaction-reverse dot blotting (PCR-RDB), real-time quantitative PCR (Realtime-PCR), Sanger sequencing, and multiplex ligation-dependent probe amplification (MLPA) (Jiang et al., 2022; Zhong et al., 2023; Wang X. et al., 2023). However, these previous approaches mainly focus on known target variants, limiting their effectiveness in identifying rarer deletions, point mutations, or novel mutations in thalassemia-related genes. Moreover, due to the distinct mutational mechanics of the HBA1, HBA2, and HBB genes, a single conventional test is often insufficient for a comprehensive assessment of variations across these globin genes (Rehman et al., 2021; Alhuthali et al., 2023). Next-generation sequencing (NGS), which is increasingly applied for diagnosing various genetic disorders, offers advantages over traditional methods in detecting thalassemia. Yang et al. (Yang et al., 2023) provided valuable population-scale NGS data on thalassemia carrier frequencies in Southern China, which further supports the necessity of broad-spectrum genotyping approaches. Cao et al. (Cao et al., 2026) observed that NGS enhanced diagnostic yield in patients with low mean corpuscular volume compared to conventional testing, with the greatest improvement seen in cases of severe microcytic hypochromic anemia. Previous studies also suggest that NGS may serve as a first-line test for genetic screening of thalassemia (Achour et al., 2021), demonstrating improved performance compared to conventional screening techniques, a notion backed by a premarital screening data from the Chinese population (Zhang et al., 2019).

In this study, we employed NGS for large-scale population screening to assess the frequency of rare thalassemia carriers in the Ganzhou region. This research marks a advancement in understanding the prevalence and variety of rare thalassemia variants in endemic areas, providing valuable insights for public health strategies. Our findings indicate that 1.29% of the screened population carried rare thalassemia variants, underscoring the necessity for targeted screening and prevention measures in regions with elevated carrier rates. Thalassemia is highly heterogeneous (Musallam et al., 2023). While some earlier studies have cautioned against the possibility of missed detections of rare thalassemia through prevention and control measures, they failed to offer specific details about the types, frequency, and distribution of rare thalassemia to enhance preventive efforts. Additionally, they did not indicate whether there are regional differences in this regard (Zhang et al., 2019; Zulkeflee et al., 2023). The Ganzhou area had a unique distribution pattern of rare thalassemia. Our findings indicated significant geographical differences in distribution. The areas of Nan Kang, Xingguo, and Zhanggong, situated to the northwest of Ganzhou, warrant increased focus in the subsequent phases of thalassemia prevention and control efforts.

Given Ganzhou’s substantial population of approximately 8,989,200 and an area spanning 39,379.64 km^2^ (https://baike.so.com/doc/3631974-3817930.html), situated in the central region of southern China, investigating the genotype and distribution of rare thalassemia in this locale is critical for establishing a theoretical foundation for effective intervention strategies. Recent research has emphasized the impact of population migration on thalassemia prevention efforts. While the prevalence of thalassemia remains high in the southern regions, the northern areas have reported significantly fewer cases (Yang et al., 2019; Li, 2017). However, as a result of population movements, the number of thalassemia patients in northern China has been rising (He et al., 2021; Zhou et al., 2021). Therefore, identifying rare thalassemia cases also holds significant implications for broader prevention and control measures, including prenatal diagnosis and genetic counseling, particularly as our findings indicate that most individuals carrying these rare variants fall within the reproductive age group. This scenario also highlights an urgent need for targeted prevention strategies for rare thalassemia in Ganzhou.

Notably, the top nine genotypes identified in rare α/β-thalassemia accounted for more than 80% of cases. Incorporating these genotypes into traditional thalassemia screening methods could represent a considerably improved strategy for detection. Some of the rare variants identified include hemoglobin variants, which have been shown to correlate with unstable hemoglobins that can lead to varying degrees of hemolytic anemia (Zhang J. et al., 2025). When combined with thalassemia, this could result in more severe manifestations of anemia (Du et al., 2021). The phenotypic expression of thalassemia is not solely determined by the presence or absence of a mutation but is modulated by multiple factors, including gene dosage, the specific nature of the variant (β^0^ vs. β^+^), co-inheritance of α- and β-thalassemia, and potentially other genetic modifiers. Furthermore, a significant genotype-phenotype discrepancy should serve as a clinical red flag, prompting further investigation. Such discrepancies may indicate the presence of additional undetected variants (e.g., rare CNVs or mutations in genes), alternative diagnoses (e.g., co-existing iron deficiency anemia or other hemoglobinopathies), or technical errors in genotyping.

Additionally, we discovered 23 novel types of thalassemia variants that had not been previously reported in databases such as HGMD, HbVar, or PubMed. According to ACMG guidelines, most of these variants were categorized as variants of uncertain significance (VUS), suggesting they are more likely benign mutations predominantly located in the 5′UTR and 3′UTR regions. Among these, six variants were classified as “Likely Pathogenic,” prompting us to collect clinical hematological data for these cases. The HBB:c.50G>T (Gly > Val) mutation (PM2_P, PP3, PM3, PM5) could be classified as a severe variant based on its association with clinical symptoms. In contrast, the phenotype associated with the HBB:c.50G>A (Gly > Asp) variant, as described in existing literature, appears to be clinically silent despite the presence of an abnormal hemoglobin band accounting for approximately 40%–45% of the total hemoglobin. (Arribalzaga et al., 1996), a discrepancy likely linked to the differing polarities of the mutated amino acids: valine (hydrophobic) versus aspartic acid (polar). In addition, several limitations warrant consideration. Our CNV detection was limited to known deletions prevalent in the Chinese population, including -α^3.7^, -α^4.2^, --^SEA^, --^THAI^, FIL, Chinese-type Gγ+(Aγδβ)^0^, Taiwanese-type, and SEA-HPFH. While this panel covers the vast majority of clinically relevant CNVs in this region, we cannot completely exclude the possibility of other rare or atypical CNVs. Importantly, any discordance between genotype and clinical phenotype would prompt further investigation using dedicated CNV detection platforms such as long-read sequencing.

In conclusion, we conducted a comprehensive study on rare and newly identified thalassemia variants in the Ganzhou area for the first time, leveraging a large sample size to explore the diversity and complexity of the condition. The enhanced diagnostic capabilities provided by NGS were essential to this research. Our findings contribute valuable data on rare thalassemia genotypes and serve as important references for future prevention and control efforts, both in this region and in other areas with high prevalence rates.

## Data Availability

The data presented in the study are deposited the Science Data Bank (ScienceDB) repository via the following private access link: https://www.scidb.cn/anonymous/WW5BVlp2.
